# Red Fruit Juice Concentration by Osmotic Distillation: Optimization of Operating Conditions by Response Surface Methodology

**DOI:** 10.3390/membranes13050496

**Published:** 2023-05-08

**Authors:** René Ruby-Figueroa, Rosanna Morelli, Carmela Conidi, Alfredo Cassano

**Affiliations:** 1 Programa Institucional de Fomento a la Investigación, Desarrollo e Innovación (PIDi), Universidad Tecnológica Metropolitana, Santiago 8940577, Chile; rruby@utem.cl; 2 Institute on Membrane Technology, ITM-CNR, Via Pietro Bucci 17/C, 87036 Rende, CS, Italy; r.morelli@itm.cnr.it (R.M.); c.conidi@itm.cnr.it (C.C.)

**Keywords:** red fruit juices, athermal concentration, osmotic distillation, response surface methodology (RSM), optimization

## Abstract

Osmotic distillation (OD) was implemented at laboratory scale to concentrate a red fruit juice produced from a blend of blood orange, prickly pear, and pomegranate juice. The raw juice was clarified by microfiltration and then concentrated by using an OD plant equipped with a hollow fiber membrane contactor. The clarified juice was recirculated on the shell side of the membrane module, while calcium chloride dehydrate solutions, used as extraction brine, were recirculated on the lumen side in a counter-current mode. The influence of different process parameters, such as brine concentration (20, 40, and 60% *w/w*), juice flow rate (0.3, 2.0, and 3.7 L min^−1^), and brine flow rate (0.3, 2.0, and 3.7 L min^−1^) on the performance of the OD process in terms of evaporation flux and increase in juice concentration, was investigated according to the response surface methodology (RSM). From the regression analysis, the evaporation flux and juice concentration rate were expressed with quadratic equations of juice and brine flow rates, as well as the brine concentration. The desirability function approach was applied to analyse the regression model equations in order to maximize the evaporation flux and juice concentration rate. The optimal operating conditions were found to be 3.32 L min^−1^ brine flow rate, 3.32 L min^−1^ juice flow rate, and an initial brine concentration of 60% *w/w*. Under these conditions, the average evaporation flux and the increase in the soluble solid content of the juice resulted in 0.41 kg m^−2^ h^−1^ and 12.0 °Brix, respectively. Experimental data on evaporation flux and juice concentration, obtained in optimized operating conditions, resulted in good agreement with the predicted values of the regression model.

## 1. Introduction

Fruits and vegetables are recognized as important sources of a number of key nutrients, including dietary fiber, folate, vitamins, minerals, and an array of bioactive substances [1]. Their consumption is associated with a reduced risk of cardiovascular disease, diabetes, cancer, and all-cause mortality [2].

Fruit juices are one of the most widely traded food products in the world. Their consumption, as well as their use as ingredients in other beverages and foods, is constantly growing owing to their valuable nutritional profile. Experimental studies clearly highlight that the consumption of 100% fruit juice is associated with higher nutrient intake and better diet quality. On the other hand, the replacement of 100% fruit juice with whole fruit equivalents had no effect on nutrient intake, except for a small increase in dietary fiber [3].

The global fruit and vegetable juice market size was valued at USD 131.62 billion in 2021 and is expected to grow at a compound annual growth rate (CAGR) of 6.3% from 2022 to 2030 [4]. Changes in consumer tastes, the adoption of healthier diets, and the COVID-19 pandemic are all factors that have had a direct or indirect impact on the global market.

Red fruits and berries have been recognized as the most important dietary sources of polyphenols such as anthocyanins, flavonols, flavan 3-ols, and benzoic and cinnamic acid derivatives. In vitro studies have reported various health effects associated with the content of anthocyanins in these fruits, including antioxidant, antimicrobial, anti-diabetic, anti-obesity, anti-inflammatory, anti-proliferative, and anti-carcinogenic activities [5,6]. Other studies confirmed that red fruit juice concentrates are an excellent source of antioxidant phenolic metabolites with marked biological activity and, therefore, suitable as ingredients for functional fruit juice mixtures and dietary supplements [7].

After their extraction from the fruit, fruit juices are either marketed at their original concentration or submitted to a concentration step. Direct or ‘not from concentrate’ (NFC) juices are obtained by pressing fresh fruits, followed by clarification, pasteurization, and packaging into containers for consumer use. These juices meet a remarkable consumer acceptance for their flavor and convenience and, more specifically, for their intrinsic properties as healthy food products. However, the extremely short shelf life of such products limits their market potential.

Fruit juice concentration is one of the basic unit operations of fruit juice processing. In this step, the solids content of the juice is increased from 10–12% up to 65–75% by the removal of water, allowing the minimization of packaging, storage, and transport costs. Additional advantages are in terms of simplification of the final juice product handling and increased stability against microbial and chemical degradation due to the reduced water activity of the juice [8]. Concentrated juices are also important ingredients in soft drinks, to which they impart significant benefits, including the nutritional and health benefits associated with fruit juice, the inclusion of natural color (in particular from red fruit juices), flavors, and clouds, and reduced costs in comparison with NFC juices. In addition, concentrated juices can be added as natural sweeteners to fruit drinks as an alternative to sucrose.

Juice concentration is traditionally accomplished by using thermal evaporation. Unfortunately, this process leads to significant losses of heat-sensitive compounds as well as sensorial characteristics such as flavor and color of the juice, which in turn impact its quality [9,10]. On the other hand, membrane filtration technologies are widely recognized and extensively used for the separation, purification, and concentration of thermosensitive compounds without thermal inputs [8]. These processes do not involve phase transitions, high temperatures, nor the use of solvents or chemical additives so preserving sensory and functional characteristics of the product. In addition, membrane processes are characterized by high selectivity based on unique separation mechanisms, low energy consumption and waste generation, easy scale-up, compactness, and modular design [11]. All these features make membrane processes efficient and clean processes that respond very well to the needs of green and sustainable technologies [12].

Among membrane processes, reverse osmosis (RO) can be used to obtain high-quality concentrated juices with high retention of nutritional, aroma, and flavour compounds [13]. However, the osmotic pressure of the juice at high concentration levels involves the use of high operating pressures, resulting in increased cost of operation and energy consumption (the osmotic pressure of a 42 °Brix pulpy orange juice is greater than 90 bar). For polymeric membranes the most efficient flux and solute recovery are at a concentration lower than 30 °Brix. Concentration polarization, fouling phenomena, and mechanical stress of RO membranes are additional drawbacks [14]. Therefore, the use of RO is suggested as a pre-concentration step before a final concentration with other technologies in order to reduce energy consumption and increase production capacity. For most polymeric membranes, the most efficient flux and solute recovery are at concentrations lower than 30 °Brix.

Compared with traditional juice concentration techniques, osmotic distillation (OD) has a great potential for concentrating fruit juices at high total soluble solids values under mild conditions of pressure and temperature, thus avoiding thermal and mechanical degradation of the product [15,16]. In this process, the driving force is the water vapour pressure difference between the two sides of a microporous hydrophobic membrane. The juice to be concentrated is recirculated on one side of the membrane, while osmotic agents, such as concentrated salt solutions, are recirculated on the other side. Water evaporates at the vapour-liquid interface of the solution with a higher vapour pressure (juice), spreads through the membrane pores, and finally condenses on the surface of the solution at the lower vapour pressure (osmotic agent) [17]. Unlike pressure-driven membrane operations, this process is characterized by several advantages, including a low fouling index, high retention of solutes, low energy consumption, and the possibility to treat solutions with high viscosity [18]. In addition, since the driving force of OD is not a hydraulic pressure difference, very high concentrations of soluble solids, above 60 °Brix, can be achieved [19,20].

The performance of OD membranes in terms of evaporation flux and juice quality has been investigated in the concentration of several fruit and vegetable juices, including apple [21,22,23], orange [22,24], pomegranate [25], noni (*Morinda citrifolia*) [26], cranberry [27], and grape [23] juices. Integrated membrane processes, including OD as the final concentration step after preliminary clarification by ultrafiltration or microfiltration and eventually preconcentration by RO, have also been investigated on both laboratory and pilot scales for apple [28], orange [19], pineapple [29], watermelon [30], kiwifruit [31], camu-camu [20], cactus pear [32], melon [33], black currant [34], red fruit [35], pomegranate [36,37], bergamot [38], and passion fruit [39] juices. All these studies confirm that evaporation fluxes can be increased after the pre-treatment of the juice by MF or UF in order to remove pulp and pectins. Bioactive compounds as well as the nutritional and sensorial characteristics of the juice are well preserved in comparison to the fresh juice, even at high levels of soluble solids (65–68 °Brix).

The main drawback of OD, when compared with RO and thermal evaporation, is its low flux (about 3 L m^−2^ h^−1^), which leads to a long treatment. Evaporation fluxes in OD are affected by several process parameters, including the type of osmotic agent, membrane pore size, concentration, flow rate, and temperature of both the osmotic agent and feed [40,41,42]. Therefore, the optimization of process parameters, which consider all interaction effects as well as main effects, is a key aspect towards a sustainable application of OD in fruit juice concentration.

Response surface methodology (RSM) is an empirical statistical technique that can be used to explore the relationship between several explanatory variables and one or more response variables [43]. Several studies have focused on the optimization of operating conditions in membrane processes by using this statistical approach [44,45,46,47]. However, few investigations have been performed until now on the effects of the main process parameters involved in the concentration of fruit juices by membrane contactors. For example, response surface models were developed by Cojocaru and Khayet [48] to predict the permeate flux and the sucrose concentration rate in the concentration of sucrose aqueous solutions by sweeping gas membrane distillation. More specifically, Onsekizoglu et al. [49] used a two-level factorial experimental design to investigate the influence of the main operating parameters on the evaporation flux and soluble solid content of apple juice concentrated by OD and membrane distillation processes.

The objective of this work was to evaluate the individual and mutual effects of juice flow rate, flow rate, and concentration of osmotic agent on the evaporation flux and total soluble solids concentration of a red fruit juice concentrated by OD using a Box-Behnken design model. In this approach, all factors are varied simultaneously over a set of experimental runs in order to determine the relationship between factors affecting the output responses of the process. Optimization of the OD process was carried out using the desirability function approach to determine the optimum operating variables.

## 2. Materials and Methods

### 2.1. Red Fruit Juice and Pre-Treatment

The red fruit juice used for the OD experiments was prepared by Citrofood Srl (Capo d’Orlando, Messina, Italy) from a blend of blood orange, prickly pear, and pomegranate juice. The original juices after extraction were mixed according to the following weight ratios: 65% blood orange juice, 25% pomegranate juice, and 10% prickly pear juice. The raw juice, after crushing and refining, was previously clarified by using a microfiltration plant equipped with two multichannel ceramic membranes with a length of 1200 mm, a pore size of 0.2 μm, and a specific filtration area of 0.42 m^2^ (Model type 19/6, supplied by Atech Innovations GmbH, Gladbeck, Germany). The characteristics of the fresh and clarified juices are shown in Table 1.

### 2.2. OD Equipment and Procedures

The clarified juice at 13 °Brix was concentrated by using a lab-scale plant supplied by ITEST s.r.l. (Corato, Bari, Italy), consisting of two independent circuits for the circulation of the solutions: one for the juice to be concentrated and the other for the concentrated brine used as an osmotic agent. Two magnetic drive gear pumps with variable motor velocities were used to recirculate the solutions on each side of the membrane. The plant was equipped with a Liqui-Cell Extra-Flow 2.5 × 8-in. membrane contactor (3M Company, Charlotte, NC, USA) containing microporous polypropylene hollow fibers with an average pore diameter of 0.2 μm and a total membrane surface area of 1.4 m^2^. The membrane contactor was connected through flexible food-grade plastic pipes to two storage tanks, one for the juice and the other for the brine, with a capacity of 5 and 10 L, respectively.

**Figure 1:**
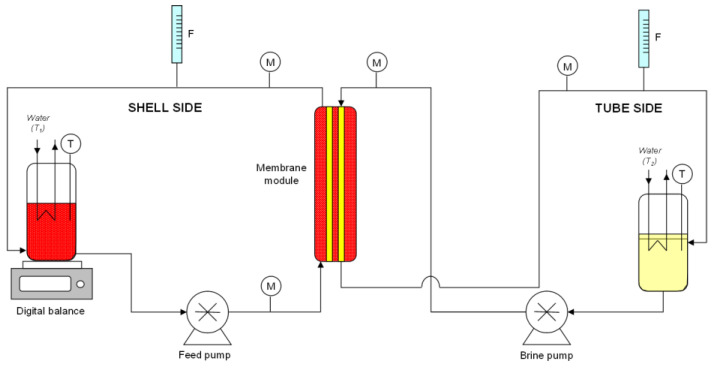
Schematic representation of the osmotic distillation plant (M, manometer; T, thermometer; F, flowmeter).

A schematic representation of the laboratory plant is depicted in Figure 1.

The clarified juice was pumped through the shell side of the membrane module while the osmotic agent was pumped in the lumen side in a counter-current mode in order to obtain the best mass transfer conditions and facilitate cleaning of the module. This configuration also allows for the minimization of the viscous effects due to the increased juice viscosity during the concentration process. Both streams were recirculated to their respective storage tanks, thus operating in total recycling mode. On average, 4 kg of pre-concentrated juice and 8 kg of brine were used for each run. This mass ratio between solutions allowed a slower dilution of the draw solution, maintaining a low value of its water activity and a relatively high driving force for the concentration process.

Calcium chloride dihydrate (from Fluka Chemie GmbH, Buchs, Switzerland) was used as an osmotic agent since it is characterized by a higher osmotic activity (ratio of its water solubility to its equivalent weight), producing a decrease of water activity greater than other salts, so allowing an increment of the driving force for the mass transfer [17,50]. For example, the activity coefficients of CaCl_2_ × 2H_2_O and NaCl solutions at 6M are 0.444 and 0.988, respectively. In this work, calcium chloride solutions with concentrations of 20, 40, and 60% *w*/*w* (corresponding to 1.7, 4.5, and 10.2 M solutions, respectively) were used.

Flow rates of feed and osmotic agents were measured with digital flow meters and varied in the range 0.3–3.7 L min^−1^ and 0.2–3.6 L min^−1^, respectively. The OD system was operated at a temperature of 25 ± 2 °C, whereas the average pressure in both compartments was about 0.6 ± 0.1 bar.

The amount of water extracted from the juice was determined gravimetrically by means of a digital balance placed under the juice container. The evaporation flux (*J_w_*), expressed as kg m^−2^ h^−1^, was then calculated according to the following equation:

(1) $$J_{w}=\frac{\Delta_{w}}{A\cdot \Delta t}$$

where Δ*_w_* is the change in juice weight observed in a defined time interval Δ*t*, and *A* is the surface area of the membrane.

The duration of each experimental run was 180 min. After each trial, the OD membrane module was cleaned according to the manufacturer’s guidelines [51]. In particular, both the tube side and the shell side were firstly rinsed with de-ionized water. Then, a KOH solution at 2% (*w*/*w*) was circulated for 1 h at 40 °C. After a short rinsing with de-ionized water, a citric acid solution at 2% (*w*/*w*) was circulated for 1 h at 40 °C. Finally, the circuit was rinsed with deionized water.

### 2.3. Experimental Design and Data Analysis

In the OD process, several parameters, including the type of osmotic agent, membrane pore size, concentration, flow rate, and temperature of both the osmotic agent and feed, affect the evaporation flux and the concentration of total soluble solids. In this study, the experimental design considered 3 variables: the concentration of the osmotic agent, the juice’s flow rate, and the osmotic agent’s flow rate. The range of selected operating variables was based on the capacity of the laboratory system.

The variable factors with the coded and actual values are presented in Table 2.

Experiments were carried out according to a Box-Behnken design composed of 15 runs, including three central points (Table 3). The correlation of the operating variables and the responses based on the Box-Behnken design were fitted to a quadratic polynomial equation using the least squares method [46]. The response variables were defined as the average evaporated flux and the soluble solid content in the juice, expressed as °Brix.

The system of equations was solved by using a multiple linear regression method [47]. The analysis of variance (ANOVA) was used to determine the linear and quadratic effects of the studied operating conditions. All the computations were performed in Statgraphics Centurion 19 (Statgraphics Technologies, The Plains, VA, USA).

## 3. Results and Discussion

The effect of studied operating variables such as brine flow rate, juice flow rate, and osmotic agent concentration on the evaporation flux and the increase in sugar content of the juice (expressed as the measurement of soluble solid content) was investigated by using a Box-Behnken design. Table 3 shows the results obtained for all the experiments performed according to this design. The linear and quadratic effects of the investigated factors and the interactions on the responses were obtained by using analyses of variance (ANOVA).

### 3.1. Effect of Operating Conditions on the Evaporation Flux

The obtained model shows a R-squared (adjusted for degree of freedom) of 98.5%, and the lack-of-fit test shows that the model is adequate for the observed data at the 95% confidence level (*p* = 0.3290). The standard error was low (equal to 0.0105), and the Durbin–Watson (DW) statistic, which is a statistical tool used to detect the presence of autocorrelation in the residuals of a regression analysis, shows that there is no indication of a serial autocorrelation in the residuals (*p* = 0.4504).

**Figure 2:**
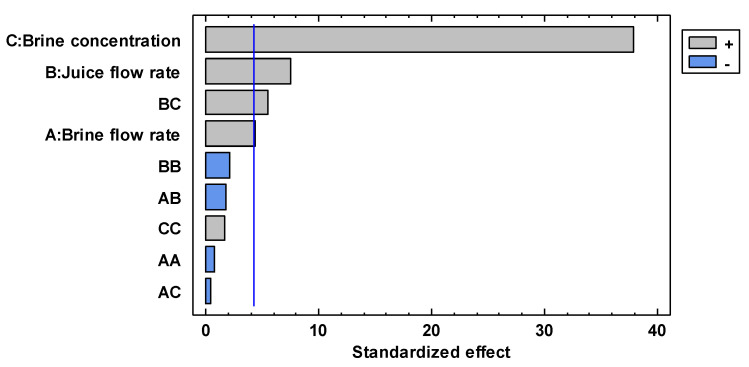
Standardized Pareto chart for the average evaporation flux.

The linear, quadratic, and interaction of each studied factor in the concentration of the red fruit juice by OD is depicted in Figure 2. According to this figure, the concentration of the osmotic agent is the most significant factor in the average evaporation flux (*p* = 0.0007), followed by the juice flowrate (*p* = 0.0173), the interaction between the juice flowrate and the concentration of the osmotic agent (*p* = 0.0313), and the brine flowrate (0.0492).

**Figure A1:**
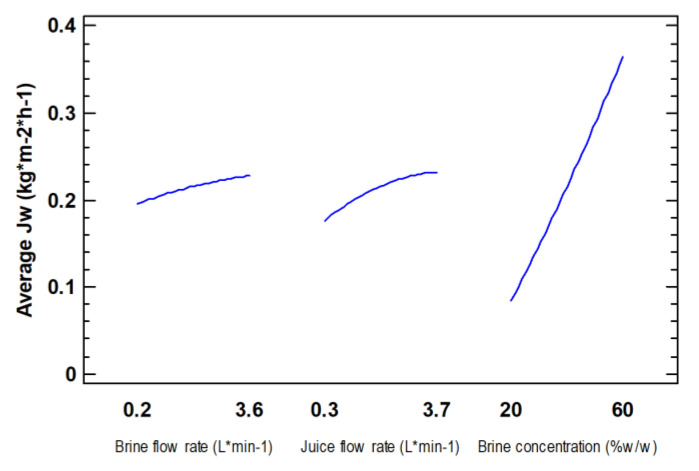
Single effect of studied operating conditions on the average evaporated flux.

The single effect of each investigated parameter on the evaporation flux is shown in Figure A1. The concentration of the extracting solution produces a significant increase in the average evaporation flux from 0.083 kg m^−2^ h^−1^ at a brine concentration of 20% *w*/*w* (1.7 mol L^−1^) until to 0.367 kg m^−2^ h^−1^ at 60% *w*/*w* (10.2 mol L^−1^). This expected result can be attributed to the increase in vapor pressure difference across the membrane with an increase in the concentration of osmotic agent solution, which in turn leads to an increased driving force for water transport through the membrane [41,52,53]. Similar effects have been reported by Rehman et al. [22] in the OD concentration of sucrose solutions at different concentrations of stripper solution (CaCl_2_ 6, 5, and 3.5 M). Similarly, Babu et al. [40] reported an increase in the transmembrane flux by increasing the concentration of the osmotic agents (NaCl or CaCl_2_) in the OD concentration of phycocyanin solution and sweet lime juice.

**Figure 3:**
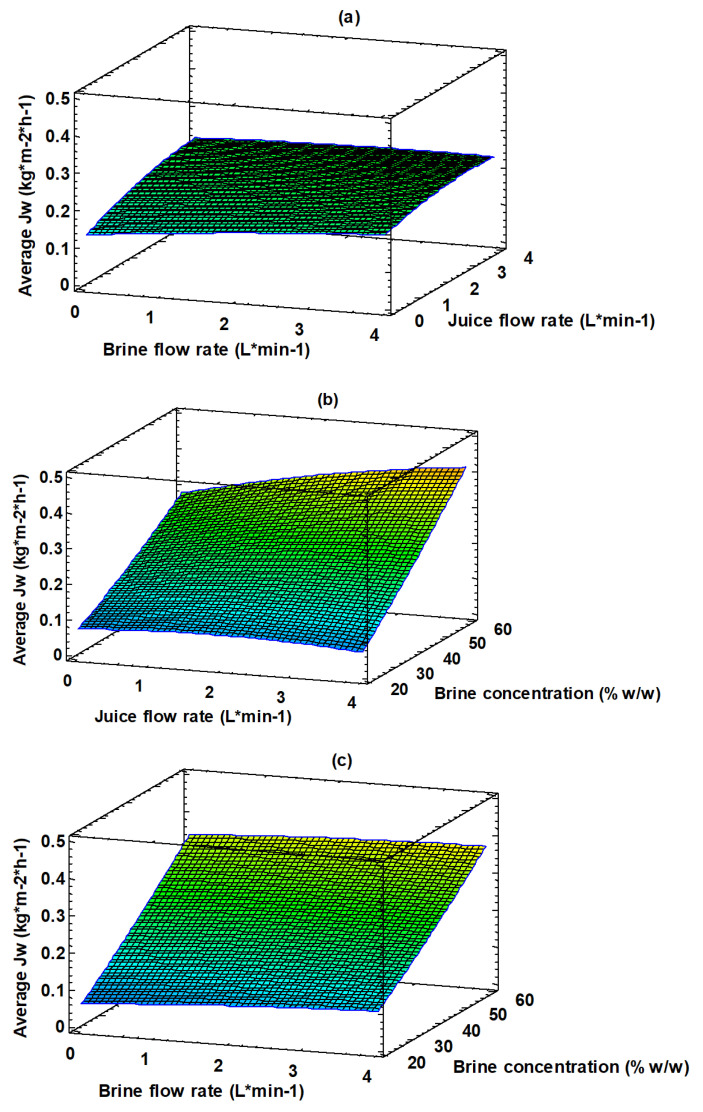
Response surface plot of evaporation flux as a function of: (**a**) juice and brine flow rate; (**b**) brine concentration and juice flow rate; (**c**) brine concentration and flow rate.

Evaporation fluxes are also influenced by feed and brine flow rates. In particular, the average evaporation flux increased by 0.034 kg m^−2^ h^−1^ when the brine flow rate was increased from 0.2 up to 3.6 L min^−1^. An increase in the average evaporation flux of 0.046 kg m^−2^ h^−1^ was observed when the feed flow rate increased from 0.3 to 3.7 L min^−1^. These effects can be attributed to the reduction in hydrodynamic boundary layer thickness, which in turn reduces the concentration polarization effect when both brine and juice velocities are increased [42,54]. However, as illustrated in Figure 3, the effect of brine concentration on the evaporation flux was significantly more prominent with respect to that of both feed and brine flow rates.

The quadratic regression equation describing the effect of the process variables on the average evaporation flux is reported as follows:

(2) $$\begin{array}{cc} Y_{1}= & -0.049\;+\;0.024X_{1}+0.004X_{2}+0.004X_{3}-0.001X_{1}^{2}-0.004X_{2}^{2}\\ & \;\;\;\;\;\;\;\;\;\;\;\;\;\;\;\;\;\;\;\;\;+0.00002X_{3}^{2}-0.003X_{1}X_{2}-0.00007X_{1}X_{3}+0.0009X_{2}X_{3} \end{array}$$

where *Y*_1_ is the predictive average evaporation flux for the OD process, *X*_1_ is the single effect of the brine flow rate, *X*_2_ corresponds to the juice flow rate effect, and *X*_3_ is the single effect of brine concentration. On the other hand, *X_i_*^2^ represents the quadratic effect of the studied factors, whereas *X_i_X_j_* represents the interaction effect.

Figure 3 shows the combined effect of the investigated variables on the average evaporation flux; the 3D plot, obtained by using Equation (2), helps to predict the response for any combination of operating variables. The response surface of the permeate flux is plotted against two operating variables, while the third variable is kept constant (level 0 in Table 2). The brine and juice flow rates have a minimum effect on the average evaporation flux (Figure 3a). On the other hand, the effect of the concentration of the osmotic agent on the evaporation flux can be appreciated in Figure 3b,c.

### 3.2. Effect of Operating Conditions on the Soluble Solid Content of the Juice

The R-squared (adjusted for degree of freedom) obtained for the soluble solids content in the juice was 97.7%. The standard error was low (equal to 0.1155), and the Durbin–Watson (DW) statistic shows that there is no indication of a serial autocorrelation in the residuals (*p* = 0.9842).

**Figure 4:**
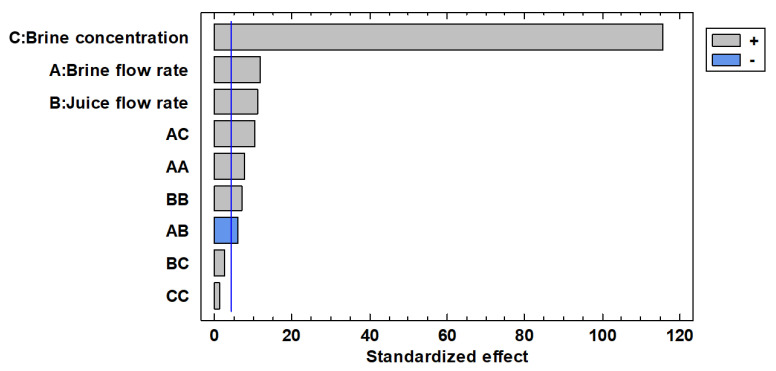
Standardized Pareto chart for the soluble solid content in the juice.

Figure 4 shows the standardized Pareto chart where the linear, quadratic, and interaction effects of each studied factor on the soluble solid content of the juice. Similar to what was observed for the average evaporation flux, the concentration of the osmotic agent is the most significant factor affecting the soluble solids content of the juice (*p* = 0.0001), followed by the brine flow rate (*p* = 0.0073) and the juice flow rate (*p* = 0.0081).

Figure 2 shows the single effect of each parameter on the soluble solid content of the juice. The concentration of the brine solution shows a significant effect on the juice concentration: it produced an increase in the soluble solids content in the juice of 10 °Brix when the system was operated at an initial brine concentration of 60% *w*/*w* (10.2 mol L^−1^). The increase in brine concentration leads to an increase in the vapour gradient across the membrane; as a consequence, higher concentrations of soluble solids in the juice are achieved in a fixed operating time [22]. On the other hand, the modification of the brine flow rate or of the juice flow rate within the tested levels produced an increase in the juice concentration of not more than 1 °Brix.

The quadratic regression equation describing the effect of the process variables on the average evaporation flux is as follows:

(3) $$\begin{array}{cc} Y_{2}= & -2.583-0.798X_{1}-0258X_{2}+0.181X_{3}+0.161X_{1}^{2}+0.144X_{2}^{2}\\ & \;\;\;\;\;\;\;\;\;\;\;\;\;\;\;\;\;\;\;+0.00017X_{3}^{2}-0.121X_{1}X_{2}+0.018X_{1}X_{3}+0.004X_{2}X_{3} \end{array}$$

where *Y*_2_ is the predictive soluble solid content in the juice for the OD filtration, *X*_1_ is the single effect of tube side rate, *X*_2_ correspond to the shell side rate effect and *X*_3_ the single effect of concentration of CaCl_2_. On the other hand, *X_i_*^2^ represent the quadratic effect of the studied factors whereas *X_i_X_j_* represent the interaction effect.

**Figure 5:**
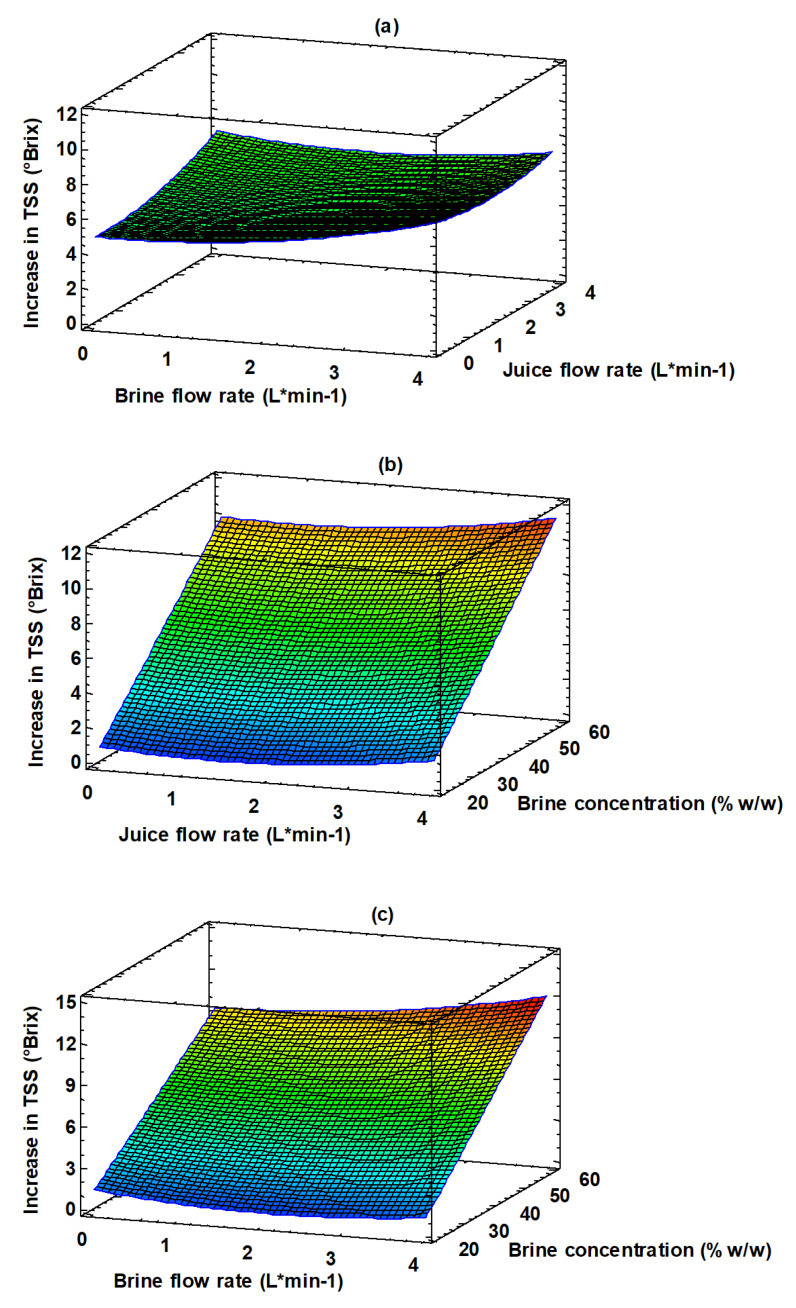
Response surface plot of TSS increase as a function of: (**a**) juice and brine flow rate; (**b**) brine concentration and juice flow rate; (**c**) brine concentration and flow rate.

Figure 5 shows the combined effect of the investigated variables on the increase of the TSS content of the juice. The 3D plot, obtained by using the Equation (3), helps to predict the response at any combination of operating variables. The response surface of the juice concentration is plotted against two operating variables, while the third variable is kept constant (level 0 in Table 2). The brine and feed flow rates had a minimum effect on the juice concentration (Figure 5a). On the other hand, the concentration effect due to the osmotic agent can be appreciated in Figure 5b,c. According to these figures the increase in the brine concentration produces an increase in the TSS from 0.8 °Brix at 20% *w*/*w* to 12 °Brix at 60% *w*/*w*.

These results confirmed that for both responses, evaporation flux and TSS content of red juice, the osmotic agent concentration was the most influential factors, followed by brine and juice flow rates. Similarly, Onsekizoglu et al. [49] found that the CaCl_2_ concentration (investigated in the range 0–65% *w*/*w*) had the predominant effect on both evaporation flux and soluble solid content of apple juice during concentration through OD, followed by the temperature difference and brine flow rate, respectively.

### 3.3. Optimization of Multiple Responses

The optimization was carried out using the desirability function (DF) in order to find the operating conditions that provide the “most desirable” responses. It is based on the conduction of experiments and fitting response models (y_k_) for all k responses, the definition of individual desirability functions for each response (d_k_), and the maximization of the overall desirability in comparison with controllable factors. For each response y_k_(x_i_), a desirability function d_k_(y_k_) assigns numbers between 0 and 1 to the possible values of y_k_, with d_k_(y_k_) = 0 representing a completely undesirable value of y_k_ and d_k_(y_k_) = 1 representing a completely desirable or ideal response value. In this work, the minimum and maximum limits used for the average evaporation flux were 0.061 (d_k_ = 0) and 0.422 (d_k_ = 1) kg m^−2^ h^−1^, respectively. In addition, the increase in the soluble solid content in the juice was 0.8 (dk = 0) and 12.0 (d_k_ = 1) °Brix for the minimum and maximum limits, respectively. The result of the optimization is shown in Table 4.

In order to validate the results obtained by the desirability function (DF), the performance of the OD process was assessed under optimum process parameters.

**Figure 6:**
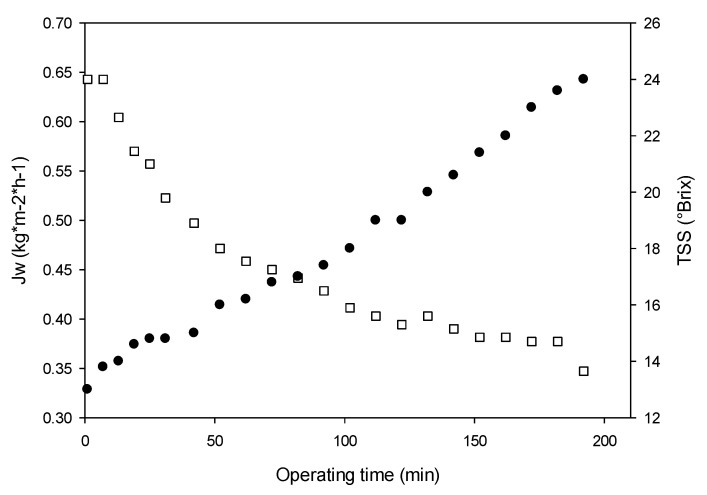
Time course of evaporation flux (white squares) and soluble solid content (black points) at optimized operating conditions during the OD of red fruit juice.

Figure 6 shows the time course of the evaporation flux and of the soluble solid content in the concentration of the clarified juice under these conditions.

In the first step of the process (range 0–70 min), the evaporation flux decreased from about 0.64 kg m^−2^ h^−1^ up to 0.45 kg m^−2^ h^−1^ (flux decline of about 30%) due to the dilution effect of the osmotic agent and, consequently, to the decrease of the driving force for water transport from the feed through the membrane. The TSS of the juice increased from 13 to 17 °Brix. In the second step (range 70–180 min), the evaporation flux declined by 22% (from 0.45 to 0.35 kg m^−2^ h^−1^), and it was accompanied by an increase of TSS up to 24 °Brix.

The average evaporation flux was 0.46 kg m^−2^ h^−1^, which is closer to that predicted by the model (0.41 kg m^−2^ h^−1^). On the other hand, the increase in the soluble solid content was 11 °Brix (from 13 °Brix up to 24 °Brix), which was closer to that predicted by the model (12 °Brix). Evaporation fluxes of the same order have been reported in the concentration of clarified pomegranate [36], cactus pear [32], kiwifruit [31], and cranberry juice [27] by using the Liqui-Cell Extra-Flow 2.5 × 8-in. membrane contactor and calcium chloride dihydrate as an osmotic agent. Onsekizoglu et al. [49] reported evaporation fluxes in the range of 0.266–1.462 kg m^−2^ h^−1^ in the concentration of apple juice by using a polypropylene capillary membrane module (MD 020 CP 2N, Microdyn, Germany) and calcium chloride dihydrate as stripping solution.

## 4. Conclusions

In this work, the response surface methodology was used to investigate the interaction of process parameters, namely juice flow rate, brine flow rate, and brine concentration, and optimize conditions for the concentration of red fruit juice by osmotic distillation with respect to evaporation flux and the concentration of soluble solids in the juice.

Optimization of multiple responses allowed us to establish the operating conditions that gave maximum productivity and the highest rate of juice concentration simultaneously. For an overall desirability of 0.985, an average evaporation flux of 0.41 kg m^−2^ h^−1^ and an increase in juice concentration of 12.0 °Brix were estimated, respectively, in optimized operating conditions of juice flow rate, brine flow rate, and brine concentration.

## Figures and Tables

**Table 1:** Composition of fresh and pre-concentrated red fruit juice (TSS, total soluble solids; TPC, total phenolic compounds; TAA, total antioxidant activity).

Parameter	Value	
	Fresh Juice	Clarified Juice
TSS (°Brix)	13.8 ± 0.27	13.0 ± 0.25
Suspended solids (%)	7.93 ± 0.15	n.d.
pH	3.59 ± 0.07	3.65 ± 0.07
Conductivity (mS/cm)	3.89 ± 0.07	4.18 ± 0.08
TPC (mg GAE L^−1^)	1370.20 ± 27.27	1341.96 ± 46.72
Flavonoids (ppm)	422.31 ± 11.80	413.33 ± 13.81
Anthocyanins (ppm)	407.99 ± 66.74	362.51 ± 15.37
TAA (mM Trolox)	11.5 ± 2.2	11.5 ± 2.2

**Table 2:** Experimental range and levels of the independent variables for the Box-Behnken design.

Parameters	Real Values of the Coded Level		
	−1	0	1
Brine flow rate (L min^−1^)	0.2	1.9	3.6
Feed flow rate (L min^−1^)	0.3	2.0	3.7
Brine concentration (% *w*/*w*)	20	40	60

**Table 3:** Experimental design and results of the Box-Behnken design.

Run	Brine Flow Rate (L min^−1^)	Juice Flow Rate (L min^−1^)	Brine Concentration (% *w*/*w*)	Average *J_w_* (kg m^−2^ h^−1^)	Increase in TSS (°Brix)
	*X*_1_	*X*_2_	*X*_3_	*Y*_1_	*Y*_2_
1	0.2 (−1)	0.3 (−1)	40 (0)	0.159	5.20
2	3.6 (1)	0.3 (−1)	40 (0)	0.200	6.00
3	0.2 (−1)	3.7 (1)	40 (0)	0.219	7.00
4	3.6 (1)	3.7 (1)	40 (0)	0.223	6.40
5	0.2 (−1)	2.0 (0)	20 (−1)	0.061	0.80
6	3.6 (1)	2.0 (0)	20 (−1)	0.108	1.40
7	0.2 (−1)	2.0 (0)	60 (1)	0.339	9.00
8	3.6 (1)	2.0 (0)	60 (1)	0.376	12.00
9	1.9 (0)	0.3 (−1)	20 (−1)	0.063	0.80
10	1.9 (0)	3.7 (1)	20 (−1)	0.075	1.20
11	1.9 (0)	0.3 (−1)	60 (1)	0.294	10.00
12	1.9 (0)	3.7 (1)	60 (1)	0.422	11.00
13	1.9 (0)	2.0 (0)	40 (0)	0.216	5.40
14	1.9 (0)	2.0 (0)	40 (0)	0.205	5.20
15	1.9 (0)	2.0 (0)	40 (0)	0.226	5.20

**Table 4:** Optimization results for the concentration of red fruit juice by OD.

Optimized coded level of variables	*X*_1_: Brine flow rate (L min^−1^)	3.32
	*X*_2_: Juice flow rate (L min^−1^)	3.69
	*X*_3_: Brine concentration (mol L^−1^)	10.2
Predicted responses	Average evaporation flux (kg m^−2^ h^−1^)	0.41
	Increase in TSS (°Brix)	12.0
Overall desirability		0.985

**Figure A2:**
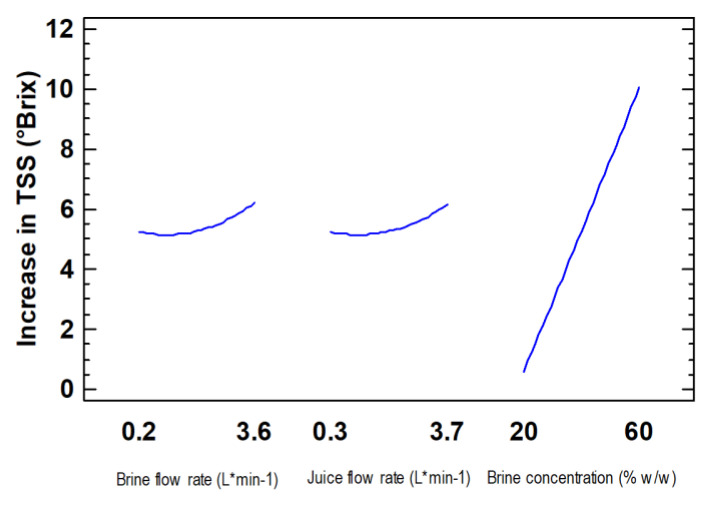
Single effect of the studied operating conditions on the soluble solid content of the juice.

## Data Availability

Data are contained within the article.
